# Supplementary material for: Major depressive episode externalizing symptoms among university students
Source: PLoS One. 2021 Jun 10;16(6):e0252027. doi: 10.1371/journal.pone.0252027 (PMC8191875; doi:10.1371/journal.pone.0252027)
Supplement: Attachment — Submitted filename: 20210423 Carta resposta Plos One.docx [file pone.0252027.s001.docx]

Dear Dr De Luca,

We appreciate the contribution of the reviewers to the article “PONE-D-20-37275 - Major depressive episode externalizing symptoms among university students”.

We have updated the article according to the requirements. Please find below (in blue) the response letter addressing all the points raised and indicating the changes applied to the article.

All the best,

Betina Flesch

Points raised and changes applied to the article:

1. Please ensure that your manuscript meets PLOS ONE's style requirements, including those for file naming. The PLOS ONE style templates can be found at

<https://journals.plos.org/plosone/s/file?id=wjVg/PLOSOne_formatting_sample_main_body.pdf> and

<https://journals.plos.org/plosone/s/file?id=ba62/PLOSOne_formatting_sample_title_authors_affiliations.pdf>

The bibliographic references, line numbers, title page and file naming were reviewed to meet PLOS ONE’s style requirements.

2. Please review your reference list to ensure that it is complete and correct. If you have cited papers that have been retracted, please include the rationale for doing so in the manuscript text, or remove these references and replace them with relevant current references. Any changes to the reference list should be mentioned in the rebuttal letter that accompanies your revised manuscript. If you need to cite a retracted article, indicate the article’s retracted status in the References list and also include a citation and full reference for the retraction notice.

The reference list was complete and correct, no papers were retracted.

3. Please amend your list of authors on the manuscript to ensure that each author is linked to an affiliation. Authors’ affiliations should reflect the institution where the work was done (if authors moved subsequently, you can also list the new affiliation stating “current affiliation:….” as necessary).

The list of authors linked to an affiliation were included in a title page of the paper.

4. We note you have included tables to which you do not refer in the text of your manuscript. Please ensure that you refer to Tables 1 and 2 in your text; if accepted, production will need this reference to link the reader to each Table.

The Tables 1 and 2 were referred in the text.

Reviewer #1: This paper is a cross-sectional survey of University students in which they were asked about their experience of externalizing symptoms of depression and this was compared against a wide range of demographic factors. It is a purely correlational study so little can be said about causal relationships. It is unfortunate that after so much work there is not a lot of information actually about the relationship between different externalizing symptoms and these demographic factors. More nuanced questioning about those symptoms would have greatly enhanced the value of the paper. The authors do note this weakness. There are a number of typographic errors (for example in Table 1 they have the caption "Curses" instead of "Courses". A quick run through to improve the English would be good although for the most part it is reasonably well written. While I think the paper could be improved it is adequate for what it intends.

The paper was reviewed by a native English speaker and typographic errors were corrected.
